# Poster Session II - A289 WITHDRAWAL OF TNF ANTAGONISTS IN PATIENTS WITH INFLAMMATORY BOWEL DISEASE IN REMISSION: A SYSTEMATIC REVIEW AND META-ANALYSIS

**DOI:** 10.1093/jcag/gwaf042.289

**Published:** 2026-02-13

**Authors:** N Ahmed, T Choudhry, M Amro Alsayed, A Khan, M Abu Zar, I Qazi, D Gnanendran, A Amrutha, A Adekunle Owolabi, H Cheema

**Affiliations:** Internal Medicine, Ain Shams University Faculty of Medicine, Cairo, Cairo Governorate, Egypt; Kettering General Hospital NHS Foundation Trust, Kettering, England, United Kingdom; Batterjee Medical College, Jeddah, Makkah Province, Saudi Arabia; Parkview Health, Fort Wayne, IN; The University of Texas Southwestern Medical Center, Dallas, TX; Internal Medicine, Baptist Hospitals of Southeast Texas, Beaumont, TX; Faculty of Health, University Hospitals Plymouth NHS Foundation Trust, UK, United Kingdom; Department of Medicine, Edmonton, AB, Canada; Department of Acute Medicine, Stockport NHS foundation trust, UK, United Kingdom; Department of Medicine, King Edward Medical University, Lahore, Pakistan

## Abstract

**Background:**

Tumor necrosis factor (TNF) antagonists are central to the management of inflammatory bowel disease (IBD), but concerns regarding long-term safety, infection risk, and costs have prompted interest in treatment de-escalation. Whether discontinuing TNF therapy in patients with sustained remission is safe remains uncertain.

**Aims:**

We aimed to conduct an updated systematic review and meta-analysis pooling all RCTs published to date on this subject in order to provide a more robust assessment of the risk of relapse, the likelihood of sustained clinical remission, and the incidence of adverse events among patients with IBD in sustained clinical remission on TNF-α antagonists. By synthesizing all available evidence, our study seeks to enhance statistical power and provide more reliable estimates to guide clinical decision-making.

**Methods:**

We performed a systematic review and meta-analysis of randomized controlled trials (RCTs) comparing TNF antagonist withdrawal with continuation in IBD patients in sustained remission. Databases including MEDLINE, Embase, CENTRAL, and ClinicalTrials.gov were searched through July 2025. Eligible trials enrolled adults with Crohn’s disease or ulcerative colitis in clinical remission. Primary outcomes were relapse risk and sustained remission. Data were pooled using a random-effects model.

**Results:**

Four RCTs comprising 485 patients were included. TNF antagonist withdrawal was associated with a significantly higher risk of relapse compared with continuation (RR: 3.00, 95% CI: 1.47–6.11). Time to relapse was also shorter in the withdrawal group (HR: 5.34, 95% CI: 2.05–13.92). Sustained clinical remission did not differ significantly between groups (RR: 0.83, 95% CI: 0.55–1.27). Withdrawal reduced infection risk (RR: 0.47, 95% CI: 0.25–0.90), while rates of gastrointestinal and serious adverse events were comparable.

**Conclusions:**

Discontinuation of TNF antagonists in IBD patients in remission substantially increases the risk and accelerates the timing of relapse, though it lowers infection risk. Careful patient selection and close monitoring are essential if withdrawal is considered.

**Funding Agencies:**

None

